# Multi-level barriers and facilitators to implementing a parenting intervention in prison, perceptions from deliverers and responsible managers: a mixed-methods study

**DOI:** 10.1186/s40359-022-00782-z

**Published:** 2022-03-24

**Authors:** Åsa Norman, Simon Swahnström, Natalia Ulfsdotter Karlström, Pia Enebrink

**Affiliations:** 1 Department of Clinical Neurosciences, Karolinska Institutet, 171 77 Stockholm, Sweden; 2 Department of Global Public Health, Karolinska Institutet, 171 77 Stockholm, Sweden; 3 Department of Psychology, Stockholm University, 106 91 Stockholm, Sweden

**Keywords:** Criminal, Child, Children of incarcerated parents, Child delinquency, CFIR, Correctional services, Crime prevention, Disadvantaged children, Implementation, Incarceration, Sweden

## Abstract

**Background:**

Children of incarcerated parents run a high risk of poor health and own delinquency and positive parenting is vital for their healthy development. Internationally, parenting interventions for incarcerated parents suggest impacts on parenting and child behaviour outcomes. The intervention For Our Children’s Sake (FOCS), was developed for incarcerated parents in Sweden and evaluated in a controlled trial with a parallel process evaluation during 2019–2021. This study constitutes part of the process evaluation and aims to describe barriers and facilitators for the implementation of FOCS, and how the intervention targets parents’ needs, as perceived by delivering group leaders and responsible correctional inspectors.

**Methods:**

In this mixed-methods study, group leaders (n = 23) and correctional inspectors (n = 12) in both intervention and control group of the FOCS trial responded to a quantitative questionnaire regarding factors of importance for intervention implementation. Group leaders (n = 12) and correctional inspectors (n = 6) in the intervention group also participated in qualitative interviews. Quantitative data were analysed using descriptive statistics and comparison of means. Qualitative data were analysed inductively using qualitative content analysis.

**Results:**

A synthesis of the quantitative and qualitative results showed that the topic of parenting and child issues in general was perceived as highly important to work with in prison, and FOCS to be an important programme in specific. At the same time, the implementation of FOCS was perceived as reliant on the individual engagement of group leaders and correctional inspectors and implementation was described as a struggle due to the scarce resources that were allowed for FOCS. Thus, additional resources and support from the Prison and Probation Service’s management were called for to facilitate implementation of FOCS, and to make it an automatic part of prison activities.

**Conclusion:**

This study showed that there was high engagement among deliverers and managers for working with parenting in prison, where the need among parents has been described as great. Additional resources and support within the overall Prison and Probation Service, is vital to facilitate implementation of FOCS and make it sustainable within the prisons. The findings can be used to refine an implementations structure for similar interventions in the prison or similar settings.

**Supplementary Information:**

The online version contains supplementary material available at 10.1186/s40359-022-00782-z.

## Introduction

Children with parents incarcerated in prison comprise a severely disadvantaged group facing an increased risk for a number of outcomes related to poor health and marginalisation, internationally [1–3], as in Sweden [2, 4, 5]. Compared to the normal child population, this child group has increased risks of poor mental health, behavioural, social, and emotional outcomes, [1, 3] and also runs a greater risk of engaging in own delinquency through an intergenerational transmission of delinquent behaviour from parent to the child [2, 5, 6].

Parents are vital in children’s development process and positive parenting, sensitivity to a child’s needs, and age adequate demands on the child, comprise essential parts of children’s healthy development [7]. Parents with a delinquent background who are being incarcerated in prison may have difficulties in practising positive parenting for various reasons such as own drug addiction, poverty, or not having much experience of positive parenting in their own childhood. Interventions focusing on family factors and positive parenting have been suggested as a measure to prevent the intergenerational effect of criminality [6, 8]. Such interventions have indicated positive influences on parent related outcomes such as parent–child interaction, parenting knowledge, empathy, parent stress, and cooperation with the other caregiver [9–11], as well as on improved child behaviours [10]. Such interventions have also suggested a possible decrease in parental recidivism [12]. All previously evaluated parenting programmes for incarcerated parents have been conducted in countries with prison and probation contexts which may have limited generalisability to Sweden. In Sweden, all prisons are run by the governmental authority The Swedish Prison and Probation Service (SPPS), which has a pronounced focus on the inmates’ rehabilitation into society during the incarceration period. Therefore, the parenting programme For Our Children’s Sake (FOCS) was developed in 2012–2014, for incarcerated mothers and fathers in prisons in Sweden with the aim to support positive parenting for children’s healthy development in. FOCS is currently being delivered as a group intervention in prisons of all security levels in Sweden. Effects of FOCS on parenting outcomes are being evaluated through a controlled trial in. The design of the evaluation has been described in a published study protocol [13]. When assessing intervention effectiveness, it is important to monitor the implementation process to gain information on factors that need revision for large-scale implementation [14]. Therefore, process evaluations benefit from exploring barriers and facilitators to intervention implementation from the perspective of several involved parties, such as deliverers, and responsible leadership. Although few previous studies that explore factors influencing the implementation of parental support interventions in prison exist, those that have been conducted have identified that factors related to recruitment and retention pf participants, the competence and engagement of programme leaders, lack of child-parent contact, appropriateness and flexibility of programme material [15], participants’ mistrust in child protection and fear of being judged or failing, own negative childhood experiences, multiple individual needs [16, 17], variation in participants’ abilities, funding and policy, and facilities in the correctional system where the programme is being carried out [18].

Process evaluations studying factors of importance for implementation benefit from theoretical guidance from implementation theory. The Consolidated Framework for Implementation Research (CFIR) comprises a framework which describes multi-level factors of importance for implementation within five overarching levels/domains (the intervention itself, the inner setting, the outer setting, characteristics of providers, and the implementation process) and provides a guidance in studies of implementation process of an intervention [19].

This study aims to describe barriers and facilitators to the implementation of the FOCS parenting programme for incarcerated parents, at intervention, personal, and different organisational levels, and how the interventions targets participants’ needs, as perceived by delivering group leaders and responsible correctional inspectors.

## Methods and materials

### Study design

This study employs a concurrent mixed-methods design using both quantitative and qualitative data collected concurrently, in order to capture a holistic view of aspects of importance for the implementation of the FOCS intervention [20]. A quantitative questionnaire was used in order to capture a broader understanding of these qualities within a larger sample of respondents, and qualitative semi-structured interviews was conducted within a smaller sample to complement the broader understanding with more in-depth information.

### Setting and the FOCS intervention

The FOCS programme was developed in 2012–2014 jointly by the SPPS and a the Swedish non-governmental organisation (Barn och ungdom med förälder i fängelse (BUFFF)). The programme targets both men and women and is carried out in group format including ten two-hour group meetings and is based on developmental psychology, attachment theory, social cognitive theory, and the Convention on the Rights of the Child (CRC) [21]. FOCS includes material in the form of a manual for group leaders, and a workbook for participants. Each session has a specific focus related to children’s experiences of parental incarceration, child development, own childhood, and parenting issues [13]. FOCS is delivered by two group leaders (GL), who take on the group leader task as part of their main employment in prison. All GLs who conduct FOCS have participated in a five-day-training, which in turn is delivered by a team of head trainers employed by the SPPS. FOCS is administered by the SPPS and includes a structure with a group leader training organisation, network of group leaders, and a coordinator for FOCS. All GLs, trainers, and the coordinator work with FOCS as part of the main employment as e.g., prison officer, counsellor etc. FOCS is included in the organisational structure within the SPSS which organises “other structured activities” together with e.g., occupational training and kitchen self-management. Each prison, led by a Prison Director, decides whether FOCS should be offered in the prison. In prisons where FOCS is offered, it is managed by a correctional inspector who is on an intermediate management level in the prison, with staff liabilities, and responsibilities for specific activities or sub-organisations within the prison, such as treatment programmes, security issues, prison work activities, or child and parenting issues. A national evaluation of the effectiveness of FOCS is being carried out in 15 prisons (8 intervention, 7 control), with three prisons for women and the remaining 12 for men.

### Participants

#### Quantitative sample

All group leaders (GL) and correctional inspectors (CI) in all prisons in the FOCS trial (intervention and control groups) were invited to participate in the quantitative data collection in order to gain a view of descriptive differences between the prisons. As allocation to intervention- and control condition in the trial was based on the operation planning at each prison, where prisons that planned for a FOCS group during the year of the trial were allocated to the intervention condition, whereas prisons planning to conduct FOCS later on were allocated to the control condition. Based on this obvious delay in planning FOCS groups in the control prisons, there may be differences related to how FOCS is prioritised between prisons in the intervention and control groups. It is therefore of interest to investigate differences related to implementation factors on intervention, personal, and different organisational levels as perceived by GLs and CIs on both groups. Of the invited 25 GLs and 13 CIs, 23 GLs, and twelve CIs responded to the questionnaire.

#### Qualitative data

All GLs and CIs in the intervention group were invited to participate in a semi-structured interview to gain in-depth information regarding implementation of the FOCS. Here, only the intervention group was invited as they had recent experiences of the intervention, thus limiting recall bias. Of the total 16 GLs and eight CIs, twelve GLs and six CIs participated in an interview. Those who did not participate did so due to sick-leave, or lack of time.

### The Consolidated Framework for Implementation Research

Quantitative and qualitative data collection in this study was guided by implementation theory in the form of the Consolidated Framework for Implementation Research (CFIR) to capture aspects influencing implementation previously identified in the health services literature that can be of relevance to the prison context. An inductive qualitative analysis allowed for additional factors to be identified in data. The CFIR [19] is a framework which describes concepts found to influence outcome which have been compiled into five overarching domains, which each includes a number of specific constructs with more detailed description of implementation factors, 39 different constructs in total [19]. Table 1 describes the CFIR domains and constructs, and the specific constructs on which items in this study were based.

**Table 1:** Description of CFIR domains, and constructs targeted by the items of the study

CFIR domains	Brief description of domains	Constructs relevant to the study
Intervention characteristics	Includes eight constructs in total which refer to adaptability to local needs, complexity, and design	• Intervention source • Relative advantage
Outer setting	Includes four constructs in total which refer to patients’ needs and resources, external policy, and incentives	• Patient needs and resources
Inner setting/the organisation	Includes 14 construct in total which refer to structural characteristics of the implementing organisation, networks and communication, culture, implementation climate, and readiness for implementation such as leadership engagement	• Readiness for implementation—leadership engagement • Culture • Readiness for implementation—available resources
Characteristics of individuals/deliverers	Includes five constructs in total which refer to knowledge/beliefs about the intervention, self-efficacy, and individual stage of change	• Individual stage of change • Knowledge and beliefs about the intervention • Self-efficacy
Process	Includes eight constructs in total which refer to the planning, execution and evaluation process	Not included in this study

### Data collection

#### Quantitative data

Quantitative data were acquired after the intervention was finished in each prison. A questionnaire capturing barriers and facilitators to the implementation of the FOCS programme in prison was distributed via a weblink to all group leaders and correctional inspectors in the intervention and control groups of the FOCS trial. The questionnaire thus provided a wide view of aspects of importance for the implementation of FOCS. To target multilevel factors that are known to influence implementation, the questionnaire was based on the CFIR [19]. No questionnaire suitable for the prison context existed at the time of the study. Therefore, a new study-specific scale was developed in a process as follows. First, a pool of items was derived based on two previous scales capturing some of the CFIR domains in different contexts [22, 23], from which items were adapted to the prison context. Second, an initial pool of 38 items was discussed within the project group and with experts on implementation research and the SPPS organization, from which items were omitted and revised to increase relevance and suitability for the target groups of GLs and CIs. Third, a revised pool of 22 items were pilot tested to elicit comprehensibility, and relevance of items. Pilot testing was conducted with three GLs and two CIs working in prisons with different security levels. Fourth, comments raised during pilot testing were discussed within the project group and experts within the field, which resulted in a final scale. The final set of items were deemed most relevant to the context and study, keeping participants’ burden in mind, thus keeping the number of items to a minimum. The final set of items comprised 16 items, which covered four of the five CFIR domains (See Quantitative results Table 3 for in-depth description of items): ‘intervention characteristics’ with two items targeting two different constructs, ‘outer setting’ with one item, ‘inner setting’ with ten items targeting three constructs, and ‘characteristics of individuals/deliverers’ with three items covering three constructs. As some items referred to specific knowledge of the FOCS intervention content, these items specifically targeted GLs or intervention group only. Thus, GLs and CIs responded to different numbers of items due to their different roles in relation to FOCS. The intervention group GLs responded to all 16 items, and CIs responded to eleven items. In the control group, GLs responded to 15 items and CIs to nine items. A response-scale from 1—disagree completely, to 5—agree completely was used for all items except two items (15 and 16, see Table 3) where a scale from 0 to 10 where 0 indicated “to a very low degree” and 10 indicated “to a very high degree” was used.

#### Qualitative data

Qualitative data were acquired after the intervention was finished in each prison through semi-structured interviews with GLs and CIs in the intervention group. Interviews were conducted by NUK (female, psychology student), SS (male, psychology student), or ÅN (female, researcher, and former prison counsellor) in Swedish, either via telephone (n = 11) or face-to-face (n = 7). Face-to-face interviews were conducted in the visiting premises of the prisons. Interview guides were developed for GLs and CIs with open-ended questions based on the five CFIR domains (Additional file 1). Interviews with GLs lasted for an average of 38 min (range: 22–56 min), and interviews with CIs lasted for an average of 27 min (range: 15–41 min). Interviews with CIs were shorter as CIs had less insight into how FOCS was conducted. Instead, interviews with CIs focused on the structure of the SPPS organisation and the specific prison where they worked, which corresponded to the CFIR domains ‘inner setting ‘, and ‘outer setting’. Interviews with group leaders covered all five CFIR domains. The qualitative data have been reported according to the Consolidated criteria for REporting Qualitative research (COREQ (Additional file 2).

### Ethical considerations

All participants provided informed consent to participate in the study. Ethical approval was obtained from the Swedish Ethical Review Authority 2019-04227.

### Data analysis

#### Quantitative

Analyses of the questionnaire data were first undertaken by investigating sub-scale reliability through internal consistency using Cronbach’s alpha. Items covering the same construct within a domain were investigated for internal consistency. Second, mean indexes for each construct covered by more than one item were calculated. Constructs covered by one item only were represented by the item mean. Further, group means were explored for each group of GLs and CIs participating in the intervention and control groups, respectively, and the total group means for GLs and CIs in the study. Lastly, group mean comparisons were explored using Mann–Whitney U test. As the group samples were small, mean comparisons were undertaken with the intention to gain a broader descriptive view of differences between groups. Comparisons were made between intervention and control groups for GLs and CIs separately, and then between GLs and CIs in total regardless of participation in intervention or control group.

#### Qualitative

Qualitative data were analysed by inductive qualitative content analysis in accordance with the procedure described by Elo and Kyngäs [31]. SS and NUK transcribed the recordings. The analysis was undertaken by ÅN, SS, and NUK, who all conducted the following steps: first, the analysts listened to the audio recordings, and read the transcripts several times to gain a holistic view of the data. Second, sections of the transcriptions that comprised data of relevance to the research aim were marked and indicated as meaning units. Third, open coding was applied to the meaning units, where each unit was labelled with a code of its core meaning. Fourth, patterns were identified among codes, and codes were merged into sub-categories in accordance with the patterns. In a fifth step, subcategories were merged into generic categories based on patterns among the sub-categories. Finally, all authors discussed and reached consensus on subthemes covering the inductive categories and the overarching theme covering all data. Data from GLs and CIs were analysed separately up until the final step of constructing the overarching theme, when all data were merged. To enhance comprehensibility of the findings the quotes include ellipses, modifications, and explanations within square brackets. When quoted in the text, participants are labelled GL and CI, with M/W to indicate sex, and a number to ensure anonymity. Data were collected in Swedish and translated into English during the final stages of analysis.

## Findings

Characteristics of the GLs and CIs who participated in the quantitative and qualitative data are found in Table 2.

**Table 2:** Characteristics of participating group leaders and correctional inspectors

Group leaders	Quantitative data (n = 23)	Qualitative data (n = 12)
Intervention group (n)	15	12
Women (n)	14	7
Education: university level (n)	11	4
Age (mean years (range))	46 (33–64)	49 (36–54)
Position (n)		
Correctional officers	18	8
Treatment staff	5	4
FOCS groups conducted	4 (0–10)	6 (1–10)

Correctional inspectors	Quantitative data (n = 12)	Qualitative data (n = 6)
Intervention group	7	6
Women (n)	7	3
Education: university level (n)	7	5
Age (mean years (range)	45 (35–57)	43 (35–57)
Work experience as CI (mean years (range))	5 (0–13)	5 (0–10)

### Quantitative findings

Table 3 displays quantitative findings related to barriers and facilitators of implementing FOCS in prison, where higher scores may indicate a facilitating construct and lower scores may indicate that the construct comprises a barrier to implementation. A significant difference (*p* = 0.005) was found between GLs and CIs regarding the *construct *“*culture*” among staff at the prison, as in staff wanting to do their best and being receptive to change, where GLs overall scored lower (mean: 3.87) than CIs overall (mean: 4.71). No other statistical group differences were detected, but a descriptive summary depicts that GLs in the intervention group (mean: 3.93) scored somewhat higher than GLs in the control group (mean: 3.29) on the *construct: leadership engagement* in the implementation of FOCS and facilitation of change at the prison. The *construct* “*available resources*” granted by the SPPS management, as in funding, personnel, and time, as well as stress in the work situation received the lowest score by both GLs and CIs, where CIs (mean: 3.24) seemed to score somewhat higher than GLs (2.96), although the difference was non-significant. All GLs (mean: 4.96) and CIs (mean: 4.92) scored rather high on the need to work on parenting in prison (*construct: *“*patients’ needs and resources*”) and all GLs in both groups and CIs in the intervention group scored high on FOCS having more advantages than disadvantages (means: 4.86–5.0, *construct: *“*relative advantage*”). All GLs and also the CIs in the intervention group scored rather high (means: 9.27–9.8) on their personally perceived importance of working with parenting and child issues in prison (*construct: *“*knowledge and beliefs*”), whereas CIs in the control group scored somewhat lower (mean: 8.8). Regarding their personal enthusiasm (means: 4.67–5.0, *construct: *“*individual stage of change*”) and the *construct: *“*self-efficacy*” (means: 8.75–8.93) towards working with parenting groups in prison both GLs and CIs scored high.

**Table 3:** Results of analysis on quantitative data, descriptive statistics, mean comparisons, and internal consistency

CFIR domain *Construct* # item	Mean (SD)						Cronbach’s Alpha
	Per intervention/control group				Total		
	GL		CI		GL	CI	
	I n = 15	C n = 8	I n = 7	C n = 5	n = 23	n = 12	
Domain: intervention characteristics							
*Construct: Intervention Source*	3.8 (1.57)	NR	4.43 (0.54)	NR	NA	NA	NA
1. I have been involved in the decision that FOCS is to be carried out in my prison^a^							
*Construct: Relative advantage*	5.0 (0.0)	5.0 (0.0)	4.86 (0.38)	NR	NA	NA	NA
2. Working with FOCS has more advantages than disadvantages^a^							
Domain: Outer setting							
*Construct: Patient needs and resources*	5.0 (0.0)	4.88 (0.35)	5.0 (0.0)	4.8 (0.45)	4.96 (0.21)	4.92 (0.29)	NA
3. There is a need to work with parenting/children's issues at the prison^a^							
Domain: Inner setting/the organisation,							
*Construct: Readiness for implementation—Leadership engagement*	3.93 (0.61)	3.29 (1.1)	NR	NR	NA	NA	0.861^c^
4. The prison management supports the implementation of FOCS in an prison in a clear and visible way^a^							
5. The prison management ensures that we have the time and space we need to discuss changes that can improve the work of the prison^a^							
6. The prison management supports change initiatives at the prison^a^							
*Construct: Culture*	3.70 (1.01)	4.19 (0.7)	4.79 (0.27)	4.6 (0.42)	3.87 (0.93)*	4.71 (0.33)*	0.8^d^
7. The staff at the prison where I work always want to do their best in their work^a^							
8. Most of the staff at the prison where I work are receptive to changing their way of working based on feedback they receive^a^							
*Construct: Readiness for implementation—available resources*	3.08 (0.87)	2.74 (0.66)	3.2 (1.05)	3.3 (0.82)	2.96 (0.81)	3.24 (0.92)	0.84^d^
9. In general, when there is a clear consensus at the various stages of the SPPS that change is necessary in the prison, we receive the necessary support from the SPPS management in the form of financial resources^a^							
10. In general, when there is a clear consensus at the various stages of the SPPS that change is necessary in the prison, we receive the necessary support from the SPPS management in the form of training^a^							
11. In general, when there is a clear consensus at the various stages of the SPPS that change is necessary in the prison, we receive the necessary support from the SPPS management in the form of human resources^a^							
12. I am too burdened and stressed to be able to do my work effectively^a^							
13. The workload in the prison adversely affects the implementation of FOCS^a^							
Domain: Characteristics of individuals/deliverers							
*Construct: Individual Stage of Change*	4.67 (0.62)	5.0 (0.0)	NR	NR	NA	NA	NA
14. I am enthusiastic about working with FOCS^a^							
*Construct: Knowledge and Beliefs about the Intervention*	9.27 (1.03)	9.75 (0.46)	9.8 (0.45)	8.8 (1.3)	9.43 (0.9)	9.3 (1.06)	NA
15. How important it is for you to work with parenting and children's issues at the prison/within the SPPS?^b^							
*Construct: Self-efficacy*	8.93 (1.91)	8.75 (1.67)	NR	NR	NA	NA	NA
16. How strong is your confidence in your ability to carry out parenting groups with inmates?^b^							

NR, not reported, i.e. the item was not included in the questionnaire to the specific sub-group of GLs/Cis; NA, not applicable (Means: no comparisons between total of groups can be made as CI responses are lacking. Cronbach’s alpha: one item only does not allow for calculation of Cronbach’s alpha)

*Significant difference between GLs and CIs at *p* < 0.05

^a^Response scale 1–5

^b^Response scale 0–10

^c^Cronbach’s alpha reported for the total of subgroup of GLs/CIs for whom the items were included in the questionnaires as Cis

^d^Cronbach’s alpha reported for the total group of GLs and CIs

### Qualitative findings

**Fig. 1:**
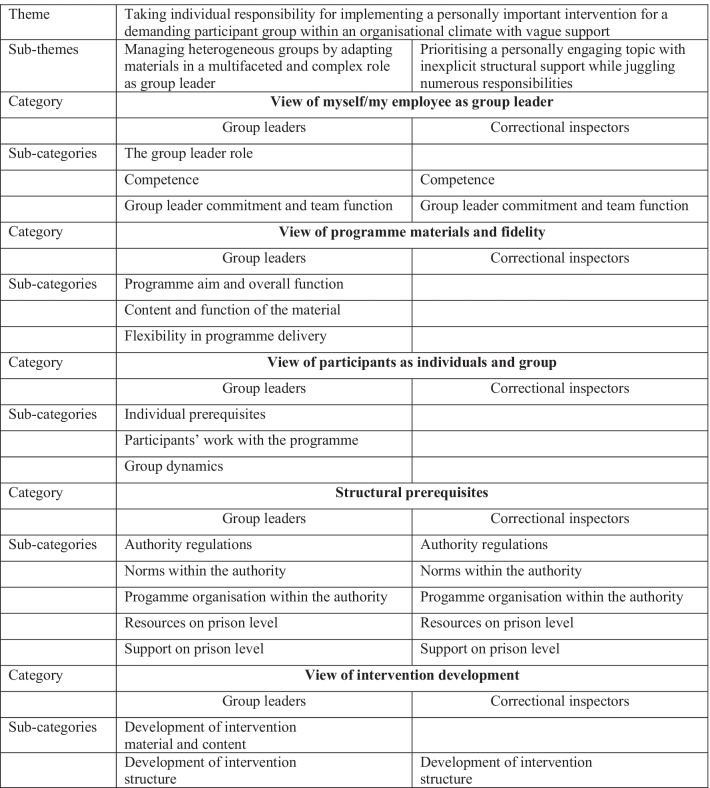
Description of qualitative findings, themes, generic categories, and sub-categories within which barriers and facilitators for the implementation of FOCS in prison are found. Group leaders and correctional inspectors are both included in the overarching theme but have separate sub-themes. Categories and sub-categories are displayed for group leaders and correctional inspectors separately. In cases where sub-categories have been found in data from both group leaders and correctional inspectors, this is indicated by the same sub-category under both participant groups. In cases where sub-categories have not been reflected in data from correctional inspectors, cells have been left blank

Qualitative findings related to barriers and facilitators to implementing FOCS in prison are depicted in Fig. 1 and in the text below in terms of the latent overarching theme and sub-themes followed by the manifest categories and sub-categories. Specified barriers and facilitators within each sub-category are listed in Additional file 3.

#### Overarching theme and sub-themes

Barriers and facilitators to the implementation of FOCS all relate to the overarching theme “Taking individual responsibility for implementing a personally important intervention for a demanding participant group within an organisational climate with vague support” which emerged in a latent synthesis of the manifest categories. The overarching theme is accompanied by one sub-theme relating to GLs: “Managing heterogeneous groups by adapting materials in a multifaceted and complex role as group leader”, and CIs: “Prioritising a personally engaging topic with inexplicit structural support while juggling numerous responsibilities”. Data revealed how GLs faced a demanding situation with a multifaceted role as both GLs and prison employees, juggling different responsibilities and relationships. Conducting FOCS put high demands on GLs’ competence and ability to handle FOCS’ topics, to be sensitive to-, and handle variation in participants’ needs, which included extreme vulnerability and antisocial attitude, on a subject which is highly sensitive to incarcerated parents: their children. The built-in programme flexibility put a lot of responsibility on the GLs’ shoulders to decide the level of fidelity, something the GLs handled differently. Also, data showed that the implementation of FOCS relied heavily on the personal engagement and prioritisation of both GLs and CIs. GLs and CIs described how they needed to work hard to implement FOCS in an environment where the overall authority structure regarding regulations and norms pays vague attention to parenting and child issues, and where the organisational affiliation of FOCS automatically puts FOCS in a low priority among prison activities, and where no specific funding for FOCS is provided to prisons, something which is key in the prioritisation of prison activities. With scarce resources, CIs felt torn between FOCS, which they found important, and other prison responsibilities which at times resulted in GLs feeling of lack of support.

#### View of myself/my employee as group leader

GLs described barriers and facilitators in relation to how their role entailed several, sometimes contrary, parts which could be demanding to handle. GLs and CIs voiced high demands on GLs’ competence to handle topics and parents’ needs, and how the FOCS’s implementation relied on GLs’ personal engagement.

##### The group leader role

GLs described barriers in terms of how they juggled different roles in group sessions as both a co-parent and in directing the group. As co-parents, GLs’ personal attitude about their own parenting, as well as being non-judgmental, and responsive to the group's susceptibility to sensitive topics, was key to a secure atmosphere and enabled sensitive discussion. In the directing role, GLs must spread the word, and direct-, and manage discussion that enabled participants to think differently.

You try to direct so that not only one person talks all the time. […] And not to accuse or condemn someone or think ‘you're doing the wrong thing’ [… And] adapt to the group. In one group I can be quite advanced and in another group I really need to tiptoe. […] And the most important thing is to listen. GL W1.

GLs also experienced dual roles in building relationships as GL, with the need to mitigate inmates’ distrust in prison staff, in and acting as correctional officers, with the need to avoid questions during sessions that violate confidentiality, and where they enforce coercive measures as a correctional officer which can be challenging to the GL-participant relationship.

Now we have this group that you build a relationship with and then you enter a different role. Where you may end up in a search case or some other case that may be a bit difficult or sensitive. How do you handle that then? GL W2.

##### Competence

GLs expressed that a broader educational background than the specific topics included in FOCS was helpful as a GL, and thus a facilitator. They also identified barriers in the need for additional competence in e.g., leadership and conflict management, to facilitate the management of specifically resistant participants, e.g., convicted for violence in close relationships. Both GLs and CIs identified a facilitator in the training that prison treatment staff have, which was perceived as specifically suitable for GLs.

The employees who have the best ability and personal suitability and skill to work with these issues is our treatment staff. They are close to these issues, they are trained in dealing with client groups, and approaching these sensitive topics. CI W1.

##### Group leader commitment and team function

Carrying out FOCS as a team where GLs can plan and support each other both during and after the sessions was perceived as a security for GLs and thus a facilitator. Both GLs and CIs identified GLs’ own interest and drive as crucial, and a facilitator for the implementation. GLs had chosen the GL assignment based on a strong interest and both GLs and CIs expressed that FOCS would not exist in a prison without GLs’ own drive as minimal additional help was available. CIs described that resources were very scarce, and GLs felt, often with frustration, that it was up to them to build a structure and argue for resources for FOCS, thus comprising a barrier for implementation.

I am passionate about parenting and children’s experiences […] At the beginning when [we] started up FOCS, there was a lot of fixing to do. We had to come up with different solutions and some things didn't work […] a help was that we were incredibly driven”. GL W3.

#### View of programme materials and fidelity

GLs described how the flexibility in the FOCS material enabled them to introduce own perceptions of functions of FOCS and to decide on how to relate to fidelity of FOCS delivery.

##### Programme aim and overall function

In addition to formal FOCS’ aim, GLs expressed own perceptions of FOCS functions which all functioned as facilitators. One such function was the focus on participants’ positive characteristics as a counterweight to the guilt and shame associated with incarceration, and another was to encourage parents to tell their children that they are in prison. Yet another function of FOCS was the potential to, in its lack of focus on personal shortcomings, motivate clients to engage in further change or to undergo treatment programmes.

The parent group can be a huge motivator for other programmes. That they realize that, "If I'm going to see my child again, I have to be drug-free." GL M1.

##### Content and function of the material

The group leaders’ manual and participants’ workbook were described as comprehensive, with clear structures which were easy to follow and facilitated the implementation of FOCS. Certain themes, e.g., regarding violence, were perceived as difficult to handle for GLs, and thus a barrier. This was particularly evident in groups with participants who were convicted for domestic violence, and for female clients with own experiences as victims.

The session around violence, there you need clarity. And that it is a session that inflicts a lot of emotions […] and there you have be prepared and a little extra alert. GL W4.

##### Flexibility in programme delivery

Group leaders related differently to fidelity to programme delivery depending on their own perception of what worked best, which seemingly functioned as both a barrier and a facilitator to the implementation of FOCS depending on how much of the core activities that were performed, and the specific needs of the group. Some GLs followed the manual strictly while others used theme titles for general discussions or as a starting point for a session based on their own preference. GLs included extra material, e.g., regarding parental cooperation, child-, or personal development, which was perceived as a facilitator to implementation. GLs also made own decisions of how to adapt sessions to a specific group, e.g., allowing discussions to deviate from the manual in the idea that it benefited the group.

But do you have to do that [follow the manual]? I follow it kind of to the subject. But then, if we don't do sessions strictly, that’s not interesting to me. […] So I usually just read what the subject is and then I usually just ‘ah okay, let's go’. Then it'll be what it will be. GL M2.

#### View of participants as individuals and group

GLs described how the great variation in participants’ backgrounds and circumstances influenced their needs of-, and engagement in FOCS, and in forming secure group dynamics.

##### Individual prerequisites

GLs described the great variation in participants’ induvial prerequisites as barriers to the implementation of FOCS. They described how participants’ variation in background and circumstances called for adaptation of themes according to the participants' needs, e.g., in relation to existing network or attitudes towards telling the child about their incarceration and about how a criminal lifestyle affects the child. Specifically, clients convicted for domestic violence were perceived to lack problem insight, and to benefit from separate FOCS groups and from completing a treatment programme before attending FOCS. Adaptations were also needed in relation to parent sex, which implies different perspectives and often degree of marginalization, but also in relation to cultural background, language skills, experience of managing an everyday life, and vulnerability with own traumatic childhood experiences.

If you were to show this [film on child perspective of violence] among all of our 80 inmates, I think more than half would recognize themselves from their own upbringing. GL W2.

##### Participants’ work with the programme

GLs described how the participants’ attitude and commitment to FOCS varied, where some participants put a lot of effort into FOCS which gave GL a drive in their work and thus facilitated implementation. Barriers consisted of participants who worked minimally with the activities, which influenced the GLs motivation negatively, and drop-out was fairly common.

We have the participants who complete the entire book the first session, to the ones go: 'Oh, I forgot it, again'. GL M3.

##### Group dynamics

GLs described barriers and facilitators related to group dynamics and how an open group dynamic created in-depth discussions, while in taciturn group themes were only discussed superficially. Barriers consisted of poor group dynamics, which resulted in a taciturn group, and was influenced by strong informal leaders whose opinions were difficult to contradict or by self-absorbed participants who repeatedly took over the discussion. Facilitators were found in a positive group dynamic, which was influenced by safety to talk openly. This, in turn, spiraled engagement in intimate and supportive discussions, with straight-forward comments between participants. A safe group dynamic, of importance to consider in the recruitment stage, was facilitated by the participants knowing each other from before the group, or by empathic, talkative individuals who catalysed the discussion.

We had participants who really opened up with thoughts and reflections. Then they engaged the others too. GL W5.

#### Structural prerequisites

GLs and CIs described how the work to implement FOCS was heavily reliant on their own engagement in an organisational environment where regulations and norms did not consider parenting, and where FOCS’s organisational affiliation provided little resources and priority.

##### Authority regulations

Regarding structural rules, serving in high security prisons caused difficulties in child- parent contact, which made certain FOCS themes difficult to implement, and thus acted as a barrier. Another barrier was found in how the recruitment or retention of a group was affected by safety rules, and sudden relocations resulted in dropouts. Furthermore, decisions for individual clients comprised a barrier to the implementation of FOCS, where GL and CIs called for greater transparency, and individual assessments as decisions for inmates were not perceived to account for parenting, which in turn affects child-parent contact. GLs described how parents’ frustration over decisions took time to debrief in FOCS sessions, and thus acted a barrier to the implementation of FOCS.

Participants want answers to questions about "why can't I call?" and "why don't I get this and that". […Y]ou have to spend at least 10–15 min each session to let them air with us GLs, who listen. And you have to do that, otherwise nothing happens [in the session]. GL W1.

##### Norms within the authority

The SPPS’s need for development regarding the work on child issues overall, and cultural perspective and gender equality in parenting specifically, was highlighted by GLs and described as barriers to the implementation of FOCS. The child perspective was described as a matter of course in female prisons as infants at times accompany their mother in prison, whereas the focus varied in male prisons. However, mothers in prison can face greater shame and a negative attitude as parents by the authority, where fathers can be praised for their small steps to make contact with their children.

There is a huge difference between female and male prisons […] Women get to have contact with their children. […] While [fathers] might get to call, that’s not a given. Plus, the SPPS does not have the same view of men and women in this regard. Here I think [the SPPS] are behind quite a bit. GL W1.

The overall lack of work with the child perspective was perceived as a barrier to FOCS as most CIs and GLs found a lack of clear directives for uniform work in prison. This lack made it each prison’s own responsibility to prioritise and implement work with the child perspective.

It could be made clearer than it is today. […] mission-wise, now it feels like everyone gets to decide for themselves how much they want to do of these [child/parent] activities. CI M1.

##### Progamme organisation wihtin the authrority

GLs and CIs voiced that FOCS’ organisational affiliation in the SPPS constituted a barrier to its implementation. They described that FOCS was not part of the SPPS treatment organisation, and thus that there were no specific assignments to carry-out FOCS, completed FOCS groups did not generate funding for prisons, and that FOCS had lower priority than treatment programmes in the SPPS, with the result that FOCS did not comprise an automatic part of prison activities.

That [FOCS] is not as high a priority as the treatment programmes. Because the prison gets money for the treatment programmes, and there’s no money in FOCS. GL M1.

CIs described it as their own choice to prioritise FOCS in the prison, but at the same that treatment programmes always came first as these generated funds. CIs called for central directives, specific FOCS assignments linked to funds so that it is not up to each prison itself to prioritize FOCS. At present, it was instead up to individuals, such as the responsible CI, to prioritise FOCS in a prison, which comprised a barrier to implementation of FOCS. Both CIs and GLs expressed that if FOCS were included in the treatment activities, it would be an automatic part of prison activities.

If there’s competition, it's the treatment programme that comes first. […] it's a money issue. If FOCS [were among] the programmes, where you get a piece of the moneybag, I also think that FOCS would have been given a different priority throughout the SPPS. […] And not dependent on the specific prison or involvement of the individual CI or GL. CI W2.

##### Resources on prison level

GLs described difficulties in allocating resources for FOCS as the basic activities in prisons are strictly divided in time and activities for inmates, resulting in many different and inflexible tasks for the staff, which comprised a barrier to implementation. FOCS sessions could be cancelled due to a lack of staff for the basic activities where a clear time plan for staff and resources facilitated implementation.

Because we work as correctional officers, we disappear as a resource. […] And during the first group, neither we nor the management had understood the consequences (laughter) or that it takes so much time. So, at times we had to cancel the group because there was actually no other staff who could work with the daily activities […] Well, next time we made a plan to cater for any time collision. GL W5.

Further barriers to implementation were found where CIs described being torn between different responsibilities where FOCS was one, but where the basic prison activities came first, and where a number of additional areas that, like FOCS, were beyond the basic activities and called for priority. The CIs described that priority for the implementation of FOCS ended up on their shoulders where they needed to argue for specific priorities that affected resource allocation in the prison. Allocating time for staff to work with FOCS was perceived as the biggest obstacle, as this always implied a resource prioritisation. This would be facilitated by linking specific financial resources to FOCS.

It’s one of many other activities we do here. [… and] on my list of different responsibilities, to be honest, FOCS is much further down the list [… where] my responsibilities are healthcare, treatment programmes and victim-offender mediation […] But it's a risk, that it depends on the people who run the groups and it depends on me, to keep this alive. […] so drive is needed, because it is not part of our base mission. CI M2.

##### Support on prison level

GLs described a supportive prison management as an important facilitator for the implementation of FOCS, especially regarding the allocation of time. Not receiving management support was described with disappointment or resignation where GLs felt left alone in the work on the child perspective, and thus as a barrier to implementation.

A facilitator was found in the CIs own perceived importance of the work with the child perspective. CIs identified their own supporting role in allocation of resources in terms of time, venue and practical support for GLs. The CIs experienced overall support from the Prison Director and other CIs, and a trust in them to pursue the FOCS work as everyone was burdened with their own responsibilities.

Support is equal from everyone. [But] I would speculate that other CIs are happy that this is being put [on me]. Because then they don't have to engage so much, knowing that I take that responsibility, […] and it's the same with the Prison Director, support is good from all levels. CI M2.

GLs found that other prison staff varied in their support where FOCS could be invisible due to high staff turnover or lack of interest. GLs identified that staff responsible for the enforcement content of an inmate must have knowledge and engagement in FOCS to enable inmates to participate. GLs found this lacking, and thus a barrier to implementation, but which could be facilitated by a structure where FOCS is planned as a clear activity.

When [the inmate] arrives in prison you plan for the activities that this particular person needs during his time in prison. And there the planners often lose out, or do not plan for FOCS and then the clients do not reach us. GL W4.

#### View of intervention development

GLs and CIs proposed extensions of FOCS to embrace the variation in parents’ needs, which would comprise facilitators to the implementation of FOCS.

##### Development of intervention material and content

GLs expressed the need for additional adaptation of themes to specific participants, e.g., clients who were convicted for domestic violence, women, or clients in high security prisons. Also, more exercises and examples could counteract discussion standstill and themes could be expanded to cover e.g., cooperation between parents and how a child perceives a parent's addiction. In addition, more information could be included in the material e.g., on children's development, attachment, and in-depth focus on how parents can support their child in practice.

It could be a little clearer about how to proceed in practice when doing certain things, how do you build a child's self-esteem? […H]ow you can actually talk more to children. GL M4.

##### Development of intervention structure

GLs expressed that group sessions could be complemented by individual participant-GL sessions, which could facilitate reflection of sensitive topics, support problem insight, and specific behavior change, in a different way than during group sessions. At the same time, individual sessions should be flexible in frequency and intensity based on participants’ needs.

[Individual sessions] are great but then you have to design it depending on the kind of [child] contact. [W]hen you sit individually, it is easier to look at your shortcomings and actually be able to say them as well. They say a little in the group but not in-depth. GL W3.

Further structural development could, similar to prison treatment programmes, include more CBT-oriented elements, and contribute to the participants’ problem insight, and increased knowledge and skills according to GLs, but CBT elements require higher GL competence. Also, a focus on parenting risks losing the current FOCS’s focus on the child perspective. GLs proposed a need for more parental interventions in prison, where FOCS, in its current form, fulfills the need to create reflection on parenthood and where an additional treatment intervention could aim at dealing with problems in parenting.

But if it's going to be a treatment programme, then I think there are quite a few more factors that are needed. […] [GLs] absolutely need more information and competence on issues to be able to deal with them […]We would absolutely need a treatment programme that targets where you have failed in your parenting. But maybe we shouldn't remove the feature of [FOCS…] to support and strengthen the parental role in a more defused way. GL W4.

CIs proposed a more fundamental perspective of everyday life e.g., an intervention focusing on overall family life with practical and emotional requirements of an adult parent. CIs also proposed a connection between FOCS and other child-parenting activities within the SPPS to provide clients with a holistic child perspective and facilitate child-parent contact.

## Discussion

This study described how group leaders and responsible correctional inspectors perceived barriers and facilitators on organisational and personal levels that influence the implementation of a parenting intervention in Swedish prisons. It also described perceptions about how the intervention met the needs of the incarcerated parents.

Together, qualitative, and quantitative findings identified that GLs and CIs found it important to work with parenting and child issues in prison in general, and that FOCS is an important programme in specific. However, additional resources and support from the SPPS management are needed to facilitate the implementation of FOCS, and to make it an automatic part of prison activities, instead of the current situation where implementation is reliant on the individual engagement of GLs and CIs in the prison and where GLs and CIs described it as a struggle to make FOCS come about despite the scarce resources allowed for FOCS. Findings from this study may be useful in guiding the implementation and sustainability of new interventions in detainment settings on the international arena, which may share high demands on staff in terms of tasks, security, and interaction with clients with high vulnerability. These settings may expand beyond the prison setting such as institutional care for substance abuse, or behavioural problems, or forensic psychiatric care.

### The importance of resources and support for successful implementation of FOCS

Findings showed that the GLs’ and CIs’ perceptions of the lack of resources influenced implementation of FOCS. In the quantitative data, the construct “available resources” in the Inner setting domain received the lowest score in the entire questionnaire from both GLs and CIs. In the qualitative data, CIs described it as a struggle to argue for the prioritisation of resource allocation to FOCS in the slim pool of resources in each prison. As FOCS does not generate specific funding for the prison the CIs described that they always had to prioritise treatment programmes before FOCS, in case of resource collision, as treatment programmes in fact generated funding for the prison. GLs pointed out that support from the prison management comprised an essential factor for successful implementation. This support could be negatively influenced by the CIs’ need to prioritise treatment programmes before FOCS, and although leadership engagement was rated and perceived as ok by GLs in both quantitative and qualitative data, there was still room for improvement according to some GLs. In addition, GLs scored significantly lower on the construct “culture” among staff at the prison, as in staff wanting to do their best and being receptive to change, which could be an indication that CIs do not register the entire atmosphere among first line correctional officer staff in the day-to-day work. CIs also perceived the available resources as somewhat higher than GLs overall, as visible in the quantitative data. They also described an understanding for the situation where it is up to themselves as responsible CIs to see to that FOCS is being implemented, as this is this case for several activities that are beyond the basic prison activities, and thus common for the role as CI. At the same time, both CIs and GLs voiced, in the qualitative data, a call for directives from the SPPS central management for FOCS to be included in a set structure with clear assignments for e.g., number of FOCS groups per year for each prison and where FOCS groups are tied to specific funding. The SPPS management’s choice not to set clear assignments tied to funding for FOCS, together with the norms and regulations that do not take parenting or child issues into regard was perceived as reflecting vague support for the parenting and child issues as seen in the qualitative findings. At the time of the data collection for this study, the CRC [21] had only just become law in Sweden, stating e.g., state parties’ responsibility for necessary care for the child’s well-being, and the child’s right to information regarding the location of a parent, which all government authorities must align with. The current CRC law in Swedish may act as a facilitator for the SPPS to expand the work on parenting and child issues. In the international field of implementation research, the importance of organisational readiness for implementation, including e.g., leadership engagement, the organisations wiliness to take on the innovation, and the innovations efficient placement within the organisational structure, has been emphasised for successful implementation of an intervention [19, 24, 25]. Specifically, resources available to carry out the intervention have been highlighted as essential to successful implementation and have also been linked to beneficial outcomes [24]. Regarding the implementation of parenting programmes in prison specifically, previous international studies have shown that resources in terms of a lack of emphasis on parenting issues in prison policies, and the prison structure with its security restrictions, sudden incidents and high demand on staff act as barriers to implementation [18, 26, 27]. Also, stable funding has been described as essential for programme sustainability [18]. Thus, in line with international evidence, findings from this study highlight that it is important to make resources available on a multitude of levels, including structural, economic, staff, levels, and to emphasise the importance of the intervention in order to facilitate implementation of a new intervention. These findings may be of relevance to a multitude of organisations and services within detainment contexts.

### The importance of working with parenting and child issues for incarcerated parents in prison, individual responsibility for implementation, and routes for extending the work

Both GLs and CIs overall reported that working with parenting and child issues for incarcerated parents is important. In the qualitative data, CIs and GLs expressed a choice to work with and prioritise FOCS based on their own interest and engagement in the topics. In the quantitative data GLs and CIs reported that there is a general need to work with these issues in the quantitative data (construct: Patient needs and resources), and that working with FOCS has more advantages than disadvantages (Construct: Relative advantage). These findings indicate helpful circumstances for successful implementation of FOCS as the constructs “relative advantage of the intervention” and “patients’ needs and resources for the intervention” have been linked to successful implementation on several occasions in previous implementation studies [24]. However, in the quantitative data, CIs in the control group scored lower on their perceived personal importance to work with FOCS than CIs in the intervention group. As the qualitative data from intervention CIs clearly reflect that the prioritisation of FOCS is the responsibility of the single CI, this lower score of control group CIs may be an indication as to why FOCS was not carried out in the control prisons at the time of the study, it may not have been prioritised and argued for by the CI, and thus not implemented and their prisons ended up in the control group. GLs in the control group also scored lower than GLs in the intervention group on the construct leadership engagement, which may support the notion that a lower personal interest and engagement of a CI may influence implementation of FOCS in the prison in a negative way. As stated above, both CIs and GLs called for a firmer structure for the implementation of FOCS in order to mitigate the deal-breaking influence of the need for personal engagement in the topic on implementation of FOCS on prison level.

Regarding the heavy personal responsibility for making FOCS a reality in prison, in the qualitative results GLs expressed that working in a team was a great support and GLs score high regarding their own self-efficacy to conduct FOCS groups in the quantitative results. However, results also showed that navigating the heterogeneous groups of parents that participate in FOCS, who often have numerous problems and troublesome backgrounds and who often mistrust and feel judged by authorities, through the often very sensitive topics in FOCS was very challenging. Both GLs and CIs called for in-depth competence to handle the sensitive topics, and interaction in the group. The high demands on GLs’ ability and competence to create a safe and non-judgemental environment to enable in-depth discussion and positive group dynamics, but also to have in-depth knowledge of the topics and profound understanding of the unique needs of the parents to facilitate programme implementation, have also been found in international studies [15, 17].The possible need for further competence enhancement among GLs could be taken into account in further implementation of FOCS specifically and similar interventions in detainment settings In addition, in our study the extensive responsibility to cater to the groups’ heterogeneity resulted in a great variation in fidelity to intervention delivery as described by the GLs themselves. The structure of the manual and supportive materials for GLs further seemed to fuel the possibility for GLs to make own decision regarding how close to the manual GLs needed to be when delivering the FOCS sessions. The balance between fidelity to programme delivery and adaptation to participants abilities and needs is a greatly explored and debated topic in the field of implementation science [19, 28]. The general idea for handling this balance is to deliver the intervention core components with high fidelity, whereas peripheral parts of the intervention can be adapted rather extensively to suit participants [28]. In order to handle this balance, GLs would need guidance regarding what comprises core and peripheral parts of FOCS. A theory of change has been proposed for the FOCS intervention [13], and a controlled effectiveness trial including the exploration of influential mediators is ongoing, which can further inform such guidance for GLs. Future intervention development for detainment settings should thoroughly consider a structure for guiding practitioners in delivering the intervention, e.g., based on a programme theory.

When emphasising the importance of working with parenting and child issues in prison, GLs and CIs proposed numerous revisions and extensions of FOCS to cater to the great need and the variation in backgrounds and prerequisites among the incarcerated parents. GLs proposed, further flexibility in the material to cater to the heterogeneity in groups, adding individual GL-parent sessions to facilitate more in-depth discussion on sensitive topics or challenges, or to include or develop yet another intervention as a complement to FOCS. A new intervention could, in addition to the child-perspective of FOCS, focus on difficulties in parenting and include more practical parenting skill training and CBT elements to enhance development of parenting skills. Also, CIs suggested more profound interventions that take an extensive perspective on adulthood and parenting responsibilities, and also a chain linking existing parenting and child interventions within the SPPS to each other for a more holistic perspective for parents and children. The different suggestions proposed by GLs and CIs have been discussed in previous international research related to the field. Interventions based on CBT have rendered substantial effects on parenting and child outcomes in the general population [29] and interventions including skills training and behavioural components have been linked to effectiveness in the specific parenting population incarcerated in prison [9]. Previous studies echo the need for programme flexibility and extension to cater to participants’ varying abilities and prerequisites, but also the need to include the often-complex background of adversity, and difficulties that incarcerated parents may have [15, 17, 18, 30]. Internationally, multilevel intervention strategies to promote and strengthen positive parenting for incarcerated parents, and to support and prevent ill-health and marginalisation in the families faced by a parent’s incarceration have been suggested as an essential need to target the multilevel adversity that these families are often facing [30, 31]. In Sweden, such chain could e.g., include child information in close connection to the arrest of a parent, parental support interventions, child support intervention, and support for child-parent contact during incarceration, child-parenting and/or family support during reunion after incarceration.

### Strengths and limitations

A strength of this study comprises the combination of qualitative and quantitative methods which provides a rich synthesis of important aspects influencing the implementation of FOCS. A further strength is the rigor and trustworthiness of the qualitative data and analysis. Data analysis included a co-coding and peer-reviewed analysis process including three researchers (ÅN, NUK, and SS), and the audit-trail of the analysis, and illustrative quotes in the results, and intersubjective agreement in the analysis process have been thoroughly described in the manuscript to ensure trustworthiness [32, 33]. Also, to ensure confirmability of the results, reflexivity journals were kept during the analysis process [33]. However, more than half of the interviews were conducted over the telephone which may limit the interaction between interviewer and participants and thus decrease richness and depth of data. Further limitations of the study comprise the small sample in the quantitative data which does not allow refined psychometric analysis for validity and reliability of the scale. Another limitation refers to that several constructs being represented by single items, which only capture one perspective of the construct. Furthermore, multiple pairwise comparison, in addition to the limitations mentioned above, suggest that the quantitative results alone, i.e., not synthesized with the qualitative results, should be interpreted with caution. Lastly, the study is limited to the perspectives of GLs and CIs only. The participants’ perspective of the implementation of FOCS will be described in a forthcoming study.

## Conclusion

This study shows that there is high engagement among deliverers and managers for working with parenting in prison, where the need among parents is described as great. Additional resources and support within the overall Prison and Probation Services, is vital to facilitate implementation of FOCS and make it sustainable within the prisons. The findings can be used to refine an implementations structure for similar interventions in the specific settings that prisons comprise.

## Supplementary Information

**Additional file 1.** Interview guide used in the semi-structured interviews to collect qualitative data.

**Additional file 2.** COREQ (COnsolidated criteria for REporting Qualitative research) Checklist for the study.

**Additional file 3.** Specified barriers and facilitators per sub-category of the qualitative findings.

## Data Availability

The datasets generated and/or analysed during the current study are not publicly available due to ethical reasons but are available from the corresponding author on reasonable request.

## Abbreviations

CI: Correctional inspector
CRC: Convention on the Rights of the Child
FOCS: For Our Children’s Sake
GL: Group leader
SPPS: Prison and Probation Services
