# Supplementary material for: Multi-level barriers and facilitators to implementing a parenting intervention in prison, perceptions from deliverers and responsible managers: a mixed-methods study
Source: BMC Psychol. 2022 Mar 24;10:79. doi: 10.1186/s40359-022-00782-z (PMC8943991; doi:10.1186/s40359-022-00782-z)
Supplement: Supplementary file 1 — Additional file 1. Interview guide used in the semi-structured interviews to collect qualitative data. [file 40359_2022_782_MOESM1_ESM.docx]

Supplementary file 1. Interview guides

**Interview guide for group leaders**

- How did you experience that "For of our children’s sake" worked in general?
- How did you perceive the function of the material?
- How do you perceive that the Prison and Probation Service treats inmates who are parents?
- How do you perceive that "For our children’s sake” " meets the need for support in parenting that inmates have?
- Before introducing "For our children’s sake”, how have you previously worked with parenting at your workplace/prison?
- In what way has the management of the prison been involved in the implementation of "For our children’s sake” in prison?
- What was the attitude of the remaining staff in prison towards "For our children’s sake”?
- What has hindered the implementation of the "For our children’s sake” in prison?
- What has facilitated the implementation of "For our children’s sake” in prison?
- Describe the process of how you became group leader of the “For of our children’s sake”?
- How did you perceive your role as being a group leader?
- What support do you need as a group leader to get started and implement For of our children’s sake”?
- What would you like to do differently if you could do "For of our children’s sake” again?

**Interview guide for correctional inspectors**

- How did you experience that "For of our children’s sake" worked in general?
- Describe the process when you first introduced “For our children’s sake” in the prison?
- How do you perceive that the Prison and Probation Service treats inmates who are parents?
- What is needed from the Prison and Probation Service to facilitate implementation of “For our children’s sake” in the prison?
- How do you perceive that "For our children’s sake” " meets the need for support in parenting that inmates have?
- Before introducing "For our children’s sake”, how have you previously worked with parenting at your workplace/prison?
- In what way have you been involved in the implementation of "For our children’s sake” in prison?
- What was the attitude of the remaining staff in prison towards "For our children’s sake”?
- What has hindered the implementation of the "For our children’s sake” in prison?
- What has facilitated the implementation of "For our children’s sake” in prison?
- What would you like to do differently if you could do "For of our children’s sake” again?
