# Supplementary material for: Multi-level barriers and facilitators to implementing a parenting intervention in prison, perceptions from deliverers and responsible managers: a mixed-methods study
Source: BMC Psychol. 2022 Mar 24;10:79. doi: 10.1186/s40359-022-00782-z (PMC8943991; doi:10.1186/s40359-022-00782-z)
Supplement: Supplementary file 3 — Additional file 3. Specified barriers and facilitators per sub-category of the qualitative findings. [file 40359_2022_782_MOESM3_ESM.docx]

Supplementary file 3. Specified facilitators and barriers within each sub-category

| **Category**  *Sub-category* | Facilitator | Barrier |
| --- | --- | --- |
| **View of myself/my employee as group leader** |  |  |
| *The group leader role* |  | Dual roles:   - Co-parent/directing group - GL/correctional officer |
| *Competence* | Broad educational background  Treatment staff training | Lack of training in:   - Leadership - Conflict management - Specific topics |
| *Group leader commitment and team function* | Working in teams  Personal interest  Personal drive | GLs’ own responsibility for implementation in prison |
| **View of programme material and fidelity** |  |  |
| *Programme aim and overall function* | Positive focus contrasting guilt and shame of incarceration |  |
|  | Encourage revelation of incarceration to the child |  |
|  | Motivate further behaviour change |  |
| *Content and function of the material* | clear structure | Emotionally challenging topics to handle for GL |
| *Flexibility in programme delivery* | Adaptation of sessions according to participants’ needs | GLs variation in fidelity of delivery |
|  | Inclusion of extra material to cater to lack in FOCS |  |
| **View of participants as individuals and as a group** |  |  |
| *Individual prerequisites* |  | Participants’ variation in background, circumstances, parent sex |
|  |  | Language skills |
|  |  | Lack of experience of managing an everyday life |
|  |  | Low problem insight |
|  |  | Antisocial attitude and behaviour |
|  |  | Negative attitude towards revealing incarceration to the child |
|  |  | High degree of marginalisation or vulnerability |
|  |  | Childhood trauma |
| *Participants’ work with the programme* | Participant engagement | Lack of participant engagement Drop out |
| *Group dynamic* | Safe atmosphere | Strong, negative leaders |
|  | Engaged participant discussions on sensitive topics | Taciturn groups |
|  | Straight-forward comments between participants | Self-absorbed talkative participants |
| **Structural prerequisites** |  |  |
| *Authority regulations* |  | High-security prison |
|  |  | Safety regulations and restrictions |
|  |  | Decisions insensitive to child/parent perspective |
| *Norms within the authority* |  | Low priority on child/parent issues |
|  |  | Sex differences in priority of child/parent issues |
|  |  | Lack of directives for prisons to work with child/parent issues |
| *Programme organization within the authority* |  | Not included in the treatment programme strucuture |
|  |  | No specific assignments to carry-out FOCS |
|  |  | FOCS does not generate funding |
|  |  | CI’s individual decision to run FOCS |
| *Resources on prison level* | Clear time plan for staff and venue | Inflexible structure for of time and activities for inmates |
|  | Funding tied to FOCS | Staff shortage |
|  |  | CI’s conflicting responsibilities |
|  |  | CI’s own heavy responsibility for implementation in prison |
| *Support on prison level* | Support from prison management | Lack of support from prison management |
|  | CI’s personal interest | Lack of FOCS knowledge among staff responsible for inmate’s’ enforcement content |
|  | Clear structure for FOCS as planned activity |  |
| **View of intervention development** |  |  |
| *Development of intervention material and content* | Adaptation and extension of themes Additional exercises Additional information |  |
| *Development of intervention structure* | Individual sessions Connecting FOCS to other child/parent activities within the PPS |  |
